# Altered Immune Regulation in Type 1 Diabetes

**DOI:** 10.1155/2013/254874

**Published:** 2013-08-21

**Authors:** András Zóka, Györgyi Műzes, Anikó Somogyi, Tímea Varga, Barbara Szémán, Zahra Al-Aissa, Orsolya Hadarits, Gábor Firneisz

**Affiliations:** ^1^Second Department of Medicine, Semmelweis University, Szentkirályi Street 46, Budapest 1088, Hungary; ^2^First Department of Obstetrics and Gynecology, Semmelweis University, Baross Street 27, Budapest 1085, Hungary

## Abstract

Research in genetics and immunology was going on separate strands for a long time. Type 1 diabetes mellitus might not be characterized with a single pathogenetic factor. It develops when a susceptible individual is exposed to potential triggers in a given sequence and timeframe that eventually disarranges the fine-tuned immune mechanisms that keep autoimmunity under control in health. Genomewide association studies have helped to understand the congenital susceptibility, and hand-in-hand with the immunological research novel paths of immune dysregulation were described in central tolerance, apoptotic pathways, or peripheral tolerance mediated by regulatory T-cells. Epigenetic factors are contributing to the immune dysregulation. The interplay between genetic susceptibility and potential triggers is likely to play a role at a very early age and gradually results in the loss of balanced autotolerance and subsequently in the development of the clinical disease. Genetic susceptibility, the impaired elimination of apoptotic **β**-cell remnants, altered immune regulatory functions, and environmental factors such as viral infections determine the outcome. Autoreactivity might exist under physiologic conditions and when the integrity of the complex regulatory process is damaged the disease might develop. We summarized the immune regulatory mechanisms that might have a crucial role in disease pathology and development.

## 1. Introduction

Type 1 diabetes (T1DM) is known to be the result of the selective damage of pancreatic *β*-cells. Other cell types of the Langerhans islets are preserved. However, the lack of insulin causes a secondary disinhibition of the glucagon-secreting *α*-cells [1]. The destruction of the *β*-cells is the consequence of a cell-mediated immune response mediated by islet-infiltrating lymphocytes and macrophages (insulitis). Cytokines secreted by macrophages are toxic for *β*-cells [2]; CD8+ cytotoxic lymphocytes are able to damage them by pore formation [3]. Autoantibodies that give the basis of clinical diagnosis are known to be secondary factors. However, promising experimental data has been published based on autoantibody neutralisation in nonobese diabetic (NOD) mice, one of the best known models of the human disease [4]. The knowledge about genetic predisposition has increased significantly in the last few decades. We know more genetic variants besides the HLA (human leukocyte antigen) alleles which are responsible for the most significant susceptibility. Although the role of genetic factors in the development of the disease is well established, by itself it seems to be insufficient to explain all the pathophysiological features of the disease. The role of numerous environmental factors is justified by experimental and epidemiological data. Nevertheless, none has been proven to be a main or generally accepted cause of the disease. More and more publications are raising the question whether autoimmunity under all circumstances would possess a definitely pathologic nature [5]. In this paper, we summarized the immunoregulatory processes including those recently put into the focus of T1DM development.

## 2. Immune Tolerance and Genetic Factors

According to twin studies, the cumulative significance of genetic features is estimated to be as high as 50 to 65% [6, 7]. Approximately 70% of type 1 diabetic carry a HLA risk allele as the strongest genetic factor. However, only 3–7% of those carrying such a HLA haplotype will ever become a patient with manifest type 1 diabetes [8]. Although NOD mice are the most commonly used models of the human disease and have high susceptibility, not all develop diabetes. However, insulitis occurs in each animal [9]. Two questions might be raised: what is necessary for the development of the insulitis? and what is necessary for its progression to diabetes? The discovery of genetic features and immunological events went on separate threads for a long time. The correlation between susceptibility and some major histocompatibility complex II (MHC-II) alleles (DR3-DQ2, DR4-DQ8) has been known to be linked to the highest (with an odds ratio—OR—of nearly seven) increase in risk [10]. Recently, some MHC-I loci were also identified among the genetic risk factors [11]. Besides the obvious role of the MHC class II and I in antigen presentation, there is an increasing number of non-HLA alleles associated with the development of type 1 diabetes. More than seventy non-HLA genes have been reported to date in the GWAStudies (genomewide association study) Catalogue based on ten large GWAScans that have variants contributing to the genetic susceptibility for T1DM with odds ratios typically below 2 (also see Table 1) [10, 12–20].

The first selection to filter autoreactive B- and T-lymphocyte clones occurs in the primary immune organs (bone marrow and thymus, resp.) where clones strongly bound to own (auto)antigens are eliminated. During the formation of the central tolerance, the autoreactive clones can be deleted or their receptor might be corrected through editing [21, 22]. Autoreactive T-cells which reach the periphery may become anergic due to being continuously flooded with self-antigens without costimulatory signals. The selection of T- and B-lymphocytes offers a mutual protection against autoimmune tendencies of each other. This is partly because B-lymphocytes are professional antigen-presenting cells (APCs), and partly because an autoreactive B-lymphocyte needs Th2 (helper T-cell, subset 2) help to expand. However, it is still possible that in some infections the pathogen-specific T-cells might be able to provide Th2 help for the autoreactive B-lymphocytes through bystander activation (as discussed later) [23]. Furthermore, in pathological conditions previously hidden self-antigens may become recognizable, leading to antigen spreading [24]. While the central tolerance is overwhelmingly genetically determined, peripheral tolerance is multifactorial.

In the selection of autoreactive T-cell clones, the antigen presentation in central lymphoid organs is crucial. Genetic background influencing the presentation of insulin in the thymus is able to weaken central immune tolerance. Those having shorter variable number tandem repeat sequences in the IDDM2 (insulin-dependent diabetes mellitus 2) locus located upstream from the insulin gene are more susceptible to autoimmune diabetes [10, 25]. In the presence of longer VNTRs, higher levels of insulin mRNA expression could be detected in the thymus. This is likely to contribute to antigen presentation with higher efficacy eventually leading to better immune tolerance [25]. When insulin expression was abrogated in the thymus using a knockout of Ins2 gene specifically in the AIRE (autoimmune regulator) expressing medullary thymic epithelial cells of mice, without affecting its expression in the beta-cells, diabetes developed in three weeks of age independent of sex [26]. 

In the central lymphoid organs, not all human antigens are present. Furthermore, after the central selection, the escape of autoreactive clones is minimal but not excluded in healthy individuals. The generation of autoreactive B-lymphocyte clones is possible even in the germinal centers through somatic hypermutation [23]. As the immune system contacts a large number of antigens during life, the optimal balance should be set in the periphery. In this process, the role of T_reg_ cells is critical. However, they do not form a homogenous population. Autoreactivity is not a binary property, even in the thymus the affinity of a T-cell to a peptide might vary on a wide range. Some of the T-cells that bind self-antigens become natural T_reg_ cells (nT_reg_) and support active immune tolerance [27]. At the same time, induced T_reg_ cells (iT_reg_) develop in the periphery from naive CD4+ T-cells. These iT_reg_ cells may have a function either in maintaining immune tolerance under tolerogenic circumstances (such as the antigens sensed in the gut mucosa under physiologic conditions) and in limiting the process in inflammation. Besides the T-cell receptor (TCR), signal transforming growth factor *β* (TGF-*β*) and interleukin 2 (IL-2) seem to be minimal requirements for iT_reg_ cell induction and also the role of APCs seems to be crucial in iT_reg_ development [28]. In this perspective, we should mention that T1DM-associated SNPs (single-nucleotide polymorphisms) were reported directly in the IL-2 [29, 30]—IL2-RA (IL-2 receptor *α*-chain, also known as CD25) [12–14] axis (see Table 1). Expression of high levels of CD25 had been the most important marker of T_reg_ cells before Foxp3 (forkhead box P3) became known. The antigen presentation will be tolerogenic and favorable for T_reg_ induction provided that the MHC-II expression on the APC is low and also the amount of costimulatory signals from APC remains limited. This can happen when there are no induction signals (as described later) for the maturation of dendritic cells (DCs). Furthermore, certain DCs are able to produce retinoic acid which is able to abrupt the effect of cytokines (such as IL-4, IFN-*γ*) that would otherwise suppress the T_reg_ inductive effect of TGF-B and IL-2, and retinoic acid eventually enhances iT_reg_ development [28, 31, 32]. Macrophages in the intestinal mucosa exhibited lower Toll-like receptor (TLR) sensitivity compared to splenic macrophages, and by IL-10 and retinoic acid production they contribute to T_reg_ induction and oral tolerance [33]. The T_reg_ cells inhibit inflammation and contribute to further T_reg_ induction via their own IL-10 and TGF-*β* secretion. Besides humoral stimuli, low cell surface CD28 and high cytotoxic T lymphocyte antigen 4 (CTLA-4) signaling promote iT_reg_ induction [34]. The binding of the T-cell surface CD28 to the B7 complex of the APC is the best known and most important costimulus in T-cell activation. CTLA-4 also binds to the B7 complex and this way abrogates this signaling. Its expression in high levels is typical of T_reg_ cells [23]. Lühder et al. could achieve prompt manifestation of T1DM in BDC2.5 TCR transgenic mice backcrossed onto the NOD genetic background by CTLA-4 inhibition which was not the result of a global T-cell activation but was caused by a more aggressive T-cell infiltration of the islets [35]. CTLA-4-deficient mice die at 2-3 weeks of age due to uncontrolled lymphoproliferation [36]. Risk polymorphisms of the CTLA-4 gene are linked to T1DM in GWAStudies [10]. CTLA-4 expression was found to be significantly lower on the mRNA level in T-lymphocytes of children with newly diagnosed T1DM [37]. Transgenic expression of an agonistic, membrane-bound single-chain anti-CTLA-4 on pancreatic *β*-cells in NOD mice could inhibit the autoimmune processes by selectively targeting CTLA-4 on pathogenic T-cells [38, 39]. The T-cell CTLA-4 expression of NOD mice was found to be diminished when examined in relation to in vitro anti-CD3 stimulation. However, the addition of exogenous IL-2 could restore the CTLA-4 expression of NOD CD8 cells to the level of healthy controls [40]. There is a clinical study in progress using low-dose IL-2 for therapeutic T_reg_ induction in T1DM [41]. The study has been completed, but not yet published.

## 3. The Janus-Character of Autoimmunity?

Although the role of B-lymphocytes is known to be secondary in T1DM, which can even develop in cases of severe congenital B-lymphocyte immunodeficiency [43], Xiu et al. could significantly delay disease onset with B-lymphocyte depletion by anti-CD20 antibody in NOD mice. They indicated that this was neither the result of T-cell reduction nor of T_reg_ induction but it was likely to be the consequence of the reduced induction of autoreactive T-cells [44]. The B-lymphocyte activating factor (BAFF, also known as tumor necrosis factor superfamily member 13b—TNFSF13B—or B-lymphocyte stimulator—BLyS) secreted by lymphoid stromal cells is necessary for B-lymphocyte survival and in cases of normal peripheric lymphocyte count autoreactive B-lymphocytes escaping central deletion might lose in the competition for BAFF. B-lymphocyte depletion using an anti-BAFF therapy in prediabetic NOD mice resulted in that the NOD mice remained diabetes free for at least 50 weeks [45]. On the other hand, transgenic overexpression of BAFF in mice resulted in the survival of autoreactive B-lymphocytes in the periphery [46]. Nevertheless, B-lymphocyte depletion might be a controversial therapy as it might delay the onset of diabetes in NOD mice, but theoretically it might also be able to enhance autoimmunity. Although B-lymphocytes as professional APCs are able to launch immune response, in vitro results suggest that they might play a regulatory role as well. In dextran sulfate sodium-induced experimental model of ulcerative colitis, Yanaba et al. described the presence of a unique B-lymphocyte population characterized by CD1d and CD5 marker positivity, which plays a regulatory role via IL-10 secretion [47]. In the coculture of primary B- and allogenic T-lymphocytes (B-lymphocytes as APCs) without additional cytokines, the expansion of T_reg_ cells characterized by Foxp3 expression was described. The partial inhibition of the MHC-II-TCR interaction enhanced this process, while CD28 stimulation by antibodies led to the generation of effector T-cells [48].

A theory first postulated by Jerne in 1974 might also be of notable significance. He indicated that the variable regions of immunoglobulins (idiotypes) may serve as antigens, and anti-idiotypic immunoglobulins can be produced against them [49]. Such a system might have a significant role in determining the intensity of immunological responses and mediating tolerance. As B-lymphocyte receptors are analogous to the immunoglobulins secreted by the cells and the B-lymphocytes present the bound antigens through MHC-II, blockade of B-lymphocyte receptors is supposed to have a profound influence on T-cell responses [50]. Furthermore, variable regions of immunoglobulins share common patterns with TCRs [51], and effective humoral immune response will not develop when there is lack of Th2 cells. Wang et al. were able to delay or, in some cases, even inhibit the onset of T1DM in NOD mice by anti-idiotypic antibodies [4]. A dysfunction of the anti-idiotypic system in autoimmunity was recently summarized by Hampe, who also cited that autoantibodies against glutamate acid decarboxylase in healthy individuals could be detected, but anti-idiotypic antibodies prevented them from binding to their target antigen [50]. 

It is described in rodents [52, 53] and also humans [54] that a shub of *β*-cell death occurs in the perinatal period. Trudeau et al. mention that in NOD mice insulitis appears approximately during the fifth week of life and never before the 15th day, subsequently following the peak of the apoptotic death wave of *β*-cells on the 13th day [53]. Theoretically, this timeframe might exist due to the immaturity of immune cells, although Höglund et al.'s finding suggests that T-cells and APCs in (their) mice are functional on the 10th day of life [55]. Trudeau et al. compared the apoptotic rate of *β*-cells in diabetes prone and resistant mice and rat strains and reported no significant difference. However, in NOD mice a higher amount of apoptotic *β*-cell remnants could be shown using the TUNEL (terminal deoxynucleotidyl transferase dUTP nick end labeling) method [53]. The dysfunction of phagocytes (predominantly macrophages) as the first islet infiltrating cells and their diminished capacity to clean apoptotic remnants might contribute to this phenomenon [56, 57]. Although apoptotic cell remnants are generally thought to have a weak potential to induce immune response, due to the impaired disposal they can become victims of secondary necrosis and encounter a stronger immunogenic potential. APCs have a crucial role in determining the nature of the antigen presentation. The inhibition of the macrophage function turned out to be protective against diabetes in NOD mice—both by the inhibition of antigen presentation and also by the diminished induction of Th1 type response (due to weaker IL-12 stimuli) [58]. In contrast, either the enhancement or the inhibition of the apoptotic rate of *β*-cells does not influence the initiation of the disease significantly [59]. On the analogy that in SLE, macrophages have an impaired apoptotic cell clearance [60], it could be hypothesized that such macrophage dysfunction may be relevant in T1DM also. As early as the 80s, it had been demonstrated that human macrophages had decreased phagocytic capacity in patients with T1DM [61]. Recently—in addition to the alternative activation of macrophages in T1DM [62]—hypopresponsivity of macrophages to IFN-*γ* has been demonstrated in the NOD mouse animal model [63], and it is possible that apoptotic cell clearance itself inhibited the macrophage responses to a major macrophage activator providing priming signal IFN-*γ* [64] and its downstream signaling (JAK-STAT1—Janus kinase and signal transducer and activator of transcription 1-path).

Turley et al. found that of the NOD and B6.H2g7 mice strains that carry the same MHC-I and MHC-II haplotype only the NOD mice develop diabetes, although in both strains potentially diabetogenic T-cells are primed [65]. Danke et al. described the presence of autoreactive T-cells in healthy individuals, but in vitro their expansion was inhibited by adding CD4+ CD25+ T-cells (T_reg_ cells) of the same individual to the culture [66]. Among others, Orban et al. have carried out a human study based on T_reg_ induction by insulin *β*-chain immunotherapy with no obvious benefit [67].

Hugues et al. could prevent NOD mice from developing diabetes by a single low dose of streptozotocin provided that *β*-cell apoptosis occurred but not in the RIP-CrmA transgenic NOD mice in which *β* cells expressed the caspase inhibitor CrmA [68]. Rayat et al. achieved similar prevention by intraperitoneal injection of in vitro streptozotocin-treated islet cells into prediabetic female NOD mice [69]. According to Hauben's review, autoimmunity is not under all circumstances a pathologic process and provided that the activation of T_reg_ cells might keep the process limited and the nature of the early immune response adequate; it may participate in the elimination of damaged tissues [5]. In BDC2.5 RAG-1-(recombination activating gene-1-) deficient mice, the generation of both B- and T-lymphocytes is impaired, and, in addition, they develop severe T1DM. The development of diabetes in the mouse model could be abrogated by the transfer of splenocytes on the 10th day of life from NOD mice, irrespective of whether the CD25 (IL-2 receptor *α*-chain) has or has not been present on the cell population [70]. Surprisingly, Balb/c mice expressing IFN-*γ* in their *β*-cells turned out to be resistant to streptozotocin-induced diabetes, while the controversial role of IL-10 (in conjunction with NOD MHC homozygosity) in autoimmune diabetes development has been suggested in experiments with transgenic mice expressing pancreatic IL-10 [71]. These data suggest that limited autoimmunity is likely to have a role in active tolerance [5], and the successful preventive attempts by supporting insulitis lead us to considering the sequence of other, for example, environmental factors.

## 4. Factors Influencing the Development of Immune Response

The existence of synergic immunological stimuli affecting separate pathways has been known for decades. However, their relevance in autoimmune diabetes is the product of recent investigation. In case of vaccination, it has been known that one-peptide antigen by itself is not always sufficient for triggering immune response. Furthermore, it is likely to induce tolerance. In general, the antigens, which are bigger and easily form aggregates, are more likely to induce immune response, while smaller soluble antigens primarily induce tolerance [72, 73]. Dresser proved in the 1960s that immunization with highly purified antigens induce immunity only in a case when an adjuvant is provided [74]. Dresser used aspecific bacterial adjuvant, but similar results could be achieved with endotoxin [75]. These observations led to the classical theory that two concurrent triggers are necessary for the appropriate induction of an immune response [73]. This basic theory by Bretscher and Cohen for B-lymphocytes [76] was later also proven in T-cell-mediated responses [77, 78]. As later clarified, during antigen presentation the APC provides numerous contact costimuli (e.g., the APC surface B7 complex binding to the surface CD28 of the T-cell) besides the MHC-II bound antigen which—together with cytokines—are necessary for T-cell activation. Janeway postulated that antigen presentation is the decision making step of the immune response, and the outcome (immunization or tolerance induction) depends on whether the APC could bind any pathogen-associated molecular pattern (PAMP) by its specific receptors (stranger hypothesis). In Janeway's theory, infections were the evolutionary drive for the immune system which was justified by the discovery of the TLRs by which the APCs detect highly conserved nonhuman (primarily bacterial and fungal) patterns. However, this theory seemed to be insufficient for the interpretation of the immune response to tumors, grafts, most viruses, and autoimmunity [73, 79]. Some of these exceptions become questionable as some viral components are ligands of intracellular TLRs through which they induce the secretion of antiviral interferons (IFN-*α* and *β*): double-stranded RNA of rota viruses is the ligand of TLR3, the single-stranded RNA of the coxsackie-B virus, probably the most often associated to T1DM, is the ligand of TLR7, while cytomegalovirus (CMV) can be sensed by the recognition of CpG DNA motif by TLR9 [80]. TLR4 functions as the receptor of bacterial lipopolysaccharide (LPS), while TLR2 recognizes cell wall components mainly from gram positive bacteria, mycobacteria, and yeast [81]. TLR2 and TLR4 are located on the cell surface but they are also involved in sensing some viruses. They primarily activate the nuclear factor kappa B (NF-*κ*B) pathway and partly alternative pathways, which results in the secretion of inflammatory cytokines [80]. *β*-cell apoptosis might participate in the priming of diabetogenic T-cells via a TLR2-dependent APC stimulation [57].

The danger hypothesis by Matzinger states that the critical signals for APCs besides the antigen are cell components released from damaged cells (danger-associated molecular patterns—DAMP) [73]. Shi et al. found that subcutaneous injection of syngenic necrotic or apoptotic cells exerts significant adjuvant effect on cytotoxic T-cell responses to ovalbumine, while the cells alone were not immunogenic [82]. Some authors describe that particles of apoptotic cells can be engulfed by macrophages and DCs, and after degradation some components are presented both on MHC-I and MHC-II complexes, hence their ability to trigger cytotoxic response [83, 84]. The properties of the antigen presentation are determined by the circumstances of the antigen-uptake: if it occurs in a “peaceful” environment, the presentation will be tolerogenic such as when apoptotic remnants are eliminated. If the appropriate factors potentiate the maturation of the DCs, it will result in T-cell activation [85].

Heat shock proteins (HSPs) are highly conserved immunogenic proteins that are involved in the pathogenesis of a variety of immune-mediated disorders, including autoimmune diseases. HSPs might serve as autoantigens, and antibodies against two epitope regions on HSP60 (AA394–413 and AA435–454) were detected in high titres in sera of children with T1DM [86]. Recently autoantibody against HSP 10 was found in sera from the majority of patients with fulminant type 1 diabetes (FT1DM) and also with autoimmune pancreatitis (AIP), and authors even suggested that an autoantibody against HSP 10 is a new diagnostic marker for both AIP and FT1DM [87]. The lymphocyte proliferative response to *Mycobacterium leprae* HSP65 of NOD mice was higher compared to their counterparts from I-E_*α*_^d^ transgenic mice that show no insulitis. In addition, splenocytes from NOD mice were able to transfer insulitis to the previously resistant transgenic strain [88]. Many cytoplasmatic components are suspected to exert adjuvant effect as DAMPs, including HSPs [73], for example, HSP70, HSP90, and HSP100 [89], which are able to stimulate the maturation of DCs [90, 91]. Certain HSPs (e.g., HSP60 and 70) generate a signal via the LPS receptor (TLR4-CD14 complex) [92], and other authors suggested that LPS-free HSP preparates were ineffective [93]. HSPs, also as carriers of peptides, are taken up by APCs and the peptide they carried can be presented on MHC-I [94]. Although a specific HSP receptor is known (CD91) [95], the TLR2/4 cluster is also involved in the uptake of HSP-peptide complexes [94]. Miyagawa et al. even indicated the role of TLR4 in binding the chemokine CXCL10, which is known to have an important role in the development of insulitis [96]. Uric acid is also able to promote the maturation of dendritic cells via binding to membrane lipids. After phagocytosis, it potentiates inflammation through enhancing IL-1*β* secretion. Uric acid is able to exert this effect only in such high concentration when crystal forming appears [97]. 

## 5. Environmental Factors and Apoptosis

According to the DIPP (diabetes prediction and prevention) study, autoantibody positivity is detectable by the age of 2 in the overwhelming majority of those who develop diabetes until the age of 10 years [98]. Therefore, the interplay between genetic susceptibility and potential triggers is likely to play a role at a very early age that gradually results in the loss of balanced autotolerance in the upcoming years and subsequently to the development of the clinical disease. Seasonal accumulation of new cases led Adams in 1926 to hypothesize the pathogenic role of viral infections. Rubella, CMV, enteroviruses, mumps, and coxsackie virus are among the most frequently suspected pathogens [99]. The potential role of coxsackie-B virus was based on the serological results of newly diagnosed T1DM patients in 1969 [100]. Later, the viral RNA was detected from the sera of such patients [101]. It is not yet clear how these viruses might be able to exert a diabetogenic effect. There is evidence that the rubella, coxsackie, and mumps viruses are able to infect *β*-cells [24], and there are experimental models for other viral infections that might lead to fulminant diabetes [102]. The similarity between human and viral proteins might offer an additional explanation, and the coxsackie-B virus indeed does contain a protein similar to the human glutamic acid decarboxylase-65 (GAD65) [24]. Experimentally, it has been proven that molecular mimicry is only able to trigger autoimmune diabetes provided that there is a full identity between the amino acid sequences as modeled in the RIP-gp (rat insulin promoter) mice which expresses a glycoprotein (gp) of the lymphocytic choriomeningitis virus (LCMV) in their *β*-cells and after an infection rapidly develop diabetes [24, 103, 104]. On the other hand, an infection with a virus containing an analogous but not identical epitope is able to promote the ongoing autoimmunity [105]. The intensity of the ongoing immune response might determine the effect of an infection [24]. When older NOD mice—in which insulitis was already present—were infected with certain coxsackie virus strains, it resulted in the speed-up of the development of diabetes [106]. However, inoculating coxsackie-B virus (CBV) into young NOD mice devoid of insulitis diminished the incidence of diabetes until the fixed endpoint. Interestingly, the more pancreatovirulent the CBV strain was, the greater the protection from T1DM onset was seen in coxsackie-B3-virus-induced pancreatitis. The immunopathology of the protection in this genetically susceptible mouse strain is not fully clarified. Authors considered that virus induced pancreatitis reveals specific host pancreatic antigens to the immune system that suppress the autoimmune insulitis in the NOD mice [106]. Recently, acceleration of murin T1DM by rotavirus was also described and associated with virus spread in regional lymph-nodes and induction of Th1-dependent antibody and cytokine response [107].

Other environmental factors such as bovine insulin containing homologous epitopes (molecular mimicry) might also boost an ongoing autoimmune process. The suspected environmental trigger bovine insulin differs only in three amino acids from human insulin. Still, the titer of antibodies against bovine insulin spontaneously decrease at the age of 12–18 months in those children in whom other diabetes-associated autoantibodies were not detected, even though they were carrying the HLA DQB1*0302 haplotype. In contrast, in those children who had at least two diabetes-associated autoantibodies at this age, the spontaneous decline in antibovine insulin antibody titer did not occur, instead the titers further increased [108].

**Figure 1:**
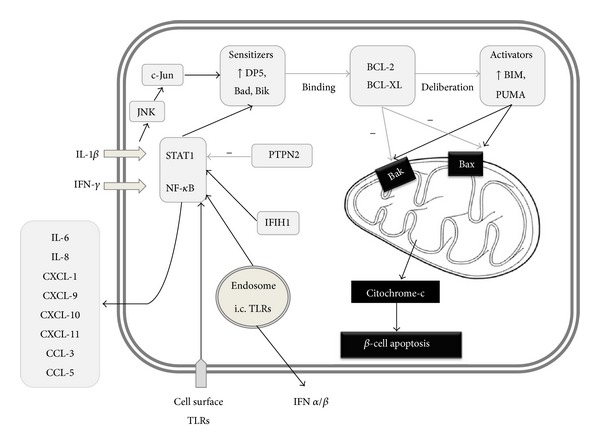
In addition to proinflammatory cytokines such as IL-1 and IFN-*γ*, the signaling via IFIH1 and various other pathogen recognition receptors mediate *β*-cell apoptotic death: upregulate certain BH3 proteins and also promote the secretion of numerous chemokines. Certain BH3 “sensitizer” proteins, for example, DP5, bind to BCL2 and BCL-XL which inhibit BAX and BAK activation and at the same time liberate the “activator” proteins (such as BIM and PUMA). PTPN2 is a negative regulator of the pancreatic *β*-cell apoptosis that reduces the BH3 protein-related apoptotic activation cascade in the *β*-cell.

The early theory that described viruses and certain environmental triggers as specific causes of T1DM via antigenic mimicry might only be sustained if taken into a more complex view. Viral infections are able to mediate the apoptotic process and some of the candidate genes identified in GWAStudies are coding proteins that are actively involved in a virus-host interplay that may—together in combination—promote the autoimmune process. The *β*-cells themselves possess pattern recognition receptors (such as IFIH1—interferon induced with helicase C domain 1—, a sensor of double-stranded viral RNA, a candidate gene [10] and TLRs) which, via the activation of NF-*κ*B and STAT1, provide proapoptotic signals for the cell [109]. The cytokines such as IFN-*γ* and IL-1*β*, which are known as main mediators of *β*-cell apoptosis, exert their effect through NF-*κ*B and STAT1 as well [109]. PTPN2 (protein tyrosine phosphatase, non-receptor type 2), which is a candidate gene according to GWAStudies has antiapoptotic activity at least in part through the blockade of JNK1 (c-Jun N-terminal protein kinase 1), which is responsible for the activation of STAT1 and BCL2L11 (BCL2-like 11, BIM; described later). Its inhibition on the translation level both in vitro (human *β*-cells) and in vivo supported IFN-induced *β*-cell apoptosis [110]. NF-*κ*B and STAT1 signaling also upregulates MHC-I expression on the cell surface, which might lead to a vicious circle by making the *β*-cell more “visible” for cytotoxic T-cells [24, 109]. Furthermore, in response to inflammatory cytokines, *β*-cells are able to express MHC-II as well [111]. Recently, many interesting data have become known on the process linking proapoptotic stimuli to the mitochondrial BAX (BCL2-associated X protein) and BAK (BCL2-antagonist/killer) activation, which is the final common path of cell death via apoptosis. In this intermediate phase, the so-called BH3 (BCL2 homologous 3) proteins have a crucial role. Based on their activity, they can be divided into two groups: the sensitizers (e.g., DP5—death protein 5—) bind to BCL2 (B-cell CLL/lymphoma 2) and BCL-XL (BCL2-like protein 1), which inhibit BAX and BAK activation, and at the same time liberate the activators (such as BIM and PUM—p53 upregulated modulator of apoptosis—) from this bond, which activate BAX and BAK [112] (Figure 1). Gurzov and Eizirik describe that in triggering this process endoplasmatic reticulum stress has an important role and in vitro stressed *β*-cells showed higher DP5 expression [112]. With DP5 inhibition on the RNA level, the apoptotic rate could be,diminished [109]. DP5 did not only have a positive effect on cell survival, but DP5 gene knockout (GKO) mice had larger *β*-cell mass and turned out to be resistant to high-fat-diet-induced glucose intolerance, directly proving the link between the immunological apoptotic and the metabolic functions [113]. Among the activators, BIM seems to be dominant. The higher apoptotic rate after PTPN2 inhibition could be significantly diminished by BIM inhibition [110]. By the inhibition of PUMA, mitochondrial BAX translocation and both apoptosis could also be diminished [114]. The combination of TNF-*α* IFN-*γ* induced DP5, PUMA, and BIM expression in human islets [115]. The figure summarizes the signaling in the *β*-cell.

The apoptotic cell remnants that might be further degraded by secondary necrosis as sources of endogenous adjuvants and inflammatory mediators might enhance the local immune response in a nonspecific manner (bystander activation) and are also able to make other previously hidden antigens available (antigenic spreading) [24]. From this view, a viral infection might be able to flare the autoimmunity up or, in the case of insufficient peripheral tolerance, even initiate an autoimmune process. The *β*-cells are not only targets but also active participants of the inflammation. Eizirik et al. analyzed the transcriptome of human *β*-cells and found that IFN-*γ* and IL-1*β* exposure resulted in several fold elevation of the chemokines CXCL-9, -10, -11, and CCL-2, -3, -5 secreted by the *β*-cell [116]. The sera of T1DM patients were shown to contain higher levels of CXCL10 compared to healthy individuals and type 2 diabetic patients [117]. The expression of CXCR3, the receptor of CXCL-9, -10, and -11 chemokines is typical of Th1 cells. Some groups were able to effectively block the manifestation of diabetes in prone mice by blocking CXCR3-linked signaling [118, 119]. However, they used the previously mentioned RIP-gp mouse, which is a perfect model of antigenic mimicry but not of the multifactorial human disease. Yamada et al. used a CXCR3 knockout NOD mouse model and expected it to be protected against diabetes. Surprisingly, the CXCR3^−/−^ NOD mouse developed diabetes even earlier, which turned out to be due to impaired navigation of T_reg_ cells to the islets [117].

## 6. Epigenetics

Although over the past fifty years there has been a (geo-epidemiologically) significant increase in incidence of T1DM, this is not in parallel with the frequency of the genetic risk. Moreover, the prevalence of the MHC-II genes responsible for approximately 40% of susceptibility has been decreased [120]. Several environmental factors may contribute to complex T1DM pathways and thus to disease manifestation. Epigenetic regulation, as a missing link, has been proposed not only to reflect the influence of environmental exposures, gender, and aging, but also to explain the discordance in monozygotic twins for the development of autoimmunity as well.

Epigenetics is a mechanism defined by mainly heritable changes of gene expression without altering directly the DNA sequence, and thus it affects genotypes to be ultimately manifested in diverse phenotypes. In general, the epigenome can be modified at three main checkpoints, like DNA methylation, posttranslational histone modifications, and expression of noncoding RNAs, such as miRNAs (micro-RNAs) and lncRNAs (long noncoding RNAs). Epigenetics plays a crucial role in the development and function of different tissues and cells, including the immune system and *β*-cell mass expansion under stress in the pancreas [121, 122]. Maturation and differentiation of immune cells, and cytokine gene expressions seem to be especially affected [123, 124]. Current lines of evidences suggest the multiple involvement of epigenetics in the pathomechanism of T1DM: epigenetic changes may influence disease outcome by affecting *β*-cell homeostasis, insulin and glucose metabolism, the gut microbiome, and immune responses.

After detecting a significant increase in *β*-cell-derived demethylated DNA in the Ins (insulin gene) yet before the onset of hyperglycemia in (prediabetic) NOD mice, Akirav et al. subsequently were able to confirm the increased demethylation of CpG sites within the insulin gene in primary human *β*-cells and also found increased levels of demethylated insulin DNA in circulating *β*-cell-derived DNA in patients with new-onset type 1 diabetes. They proposed this observation as an alteration contributing to T1DM pathology, as well as a potentially noninvasive approach for detecting in vivo *β*-cell death [125].

By using the chromatin immunoprecipitation linked to microarray (ChIP-chip) approach to compare genome wide histone H3 lysine 9 dimethylation (H3K9me2) patterns in peripheral blood lymphocytes and monocytes from T1DM patients, the T1DM candidate gene CTLA4 has been displayed higher H3K9me2 at the promoter region yet standing as an example of interface between genetic and epigenetic information in T1DM [126].

Miao et al. observed marked variations in H3K9-acetylation (H3K9Ac)—that is associated with promoters and active genes—levels at the upstream regions of HLA-DRB1 and HLA-DQB1 in T1DM monocytes and also demonstrated increased expression of HLA-DRB1 and HLA-DQB1 on monocytes in response to interferon and TNF treatment that were accompanied by changes in H3K9Ac at the same promoter regions as those seen in the patients' cells. Therefore, they suggest that the H3K9Ac status may regulate the transcriptional response of HLA-DRB1 and HLA-DQB1 to cytokine stimuli [127].

The complexity of epigenetic mechanisms is well characterized by the recent finding that the promoter of the Ins (insulin) gene is part of an extended “open” chromatin domain and as such is in physical contact with the Syt8, a gene that is located at 300 kb distance in the genome, and interestingly this contact between Ins and Syt8 is strengthened by glucose in pancreatic islets [128]. 

Certain miRNAs are also related to T1DM: miR-21a and miR-93 were shown to be downregulated in peripheral blood mononuclear cells of T1DM patients. Moreover, a population of T_reg_ cells of T1DM patients showed a higher expression of miR-146 that is crucial in maintaining the suppressor function of T_reg_ cells, while a lower expression of eight other miRNAs (20b, 31, 99a, 100, 125b, 151, 335, and 365) were found [129, 130]. 

Furthermore, a role for long noncoding (lnc) RNAs, both in the *cis* and *trans* regulation of transcription via interaction with chromatin modifying complexes to target epigenetic marks to particular genomic loci, has only been recently described. An islet-specific lncRNA expressed from the Pdx1 locus regulates Pdx1 activity, a master gene of *β*-cell differentiation and regulation, therefore has a potential impact on maintaining glucose homeostasis. Long noncoding RNA molecular studies might open a yet largely unrevealed novel layer of transcribed but not translated genetic information and new dimensions in diabetes research [131–134].

## 7. Summary

Type 1 diabetes mellitus is a prototype disorder of both endocrine and organ-specific autoimmune diseases. There is a growing amount of evidence suggesting that autoimmunity cannot be interpreted as a binary state. It rather becomes pathologic through impaired regulation. From the view of the congenital susceptibility, the role of environmental factors might be revised as some might be considered rather enhancers than triggers. The early and adequate nature of the response with optimal limitation of autoimmunity might be the difference between the physiologic and the pathologic conditions [5]. The impaired elimination of apoptotic remnants may create a less tolerogenic inflammatory environment favorable for the initiation of autoimmunity [53]. The transient local inflammation due to, for example, a viral infection in combination with the impaired immune regulatory functions that are in part determined by the genetic background may eventually lead to the generation and expansion of autoreactive T-cells [24, 135]. Data from the World Health Organization Diabetes Mondiale (WHO Diamond) Project suggested the role of late enhancers: as the later the disease onset was, the more the pronounced seasonality evaluators could find [136]. Therefore, the possibility of an early immunization as a preventive approach might be raised.

GWAStudies made real breakthrough in identifying non-HLA genetic variations that participate in the establishment of the genetic susceptibility. In addition, the identification of a number of candidate genes and their risk polymorphisms GWAS contributed significantly to the understanding of the molecular pathology of certain immune mechanisms. Epigenetics has also been reported to contribute significantly to the pathology of this autoimmune disease.

The most recent national and international epidemiologic data still show elevation in T1DM incidence in all age groups but especially among the youngest, under 4 years [137, 138]. Although the role of certain viral infections cannot be excluded in the priming, such epidemics have become even less frequent, which is the theoretical basis of the hygiene hypothesis. Yet, a germ-free environment might not only exclude an immunomodulatory effect of various infections [139], but also, if the time of the first infections is delayed, it may more easily enhance latent autoimmunity. Interactions of metabolic and immunologic processes are also likely to be considered as between potential cause of the epidemiological tendencies. According to the results of the search for diabetes in the youth (USA), 35.4% of those diagnosed with diabetes under the age of 20 are insulin resistant, and 19.5% also have autoantibodies [140]. 

A number of clinical trials have been conducted or are still under clinical investigation, and the framework of this review limited us to comprehensively report all trials. Effector and regulatory T-cells are known to differ in their affinity to IL-2 and therefore theoretically provide a narrow range where T_reg_ induction by IL-2 is feasible without the induction of effector T-cells (as summarized earlier) [41]. Induction of immune tolerance via nasal, or oral whole insulin antigen, intramuscular insulin B-chain and subcutaneous GAD65 (with alum) administration or subcutaneous hsp60_437–460_ (p277) administration either failed to demonstrate clinically significant improvement or resulted in controversial outcomes [141]. Studies administering D-vitamin based on its immunomodulatory properties have also failed to demonstrate clear clinical benefit [142, 143]; nevertheless, a recent study found that T1DM risk was highest among individuals whose 25(OH)D vitamin levels were in the lowest 20% of those measured and concluded that low 25(OH)D-vitamin levels may predispose young adults to the development of T1DM [144].

Our study group have reported earlier that the dipeptidyl-peptidase-4 (DPP-4)-incretin axis might be dysregulated and the serum DPP-4 enzymatic activity is higher in patients with T1DM than in healthy controls [145]. Blandino-Rosano et al. found that in vitro GLP-1 (glucagon-like peptide-1) is able to reverse the inhibition of extracellular signal-regulated kinase 1 and 2 (ERK1 and 2) phosphorylation and the *β*-cell antiproliferative effect of proinflammatory cytokines IL-1*β*, IFN-*γ*, and TNF-*α* [2]. In addition to the *β*-cell protective effect of GLP-1, the incretin agonists might have a role in the maintenance of the peripheral T_reg_ cell population [146]. The results of a few pilot studies indicated a lower amount of insulin needed to gain similar (or even better) glycemic control using a DPP-4 inhibitor or a GLP-1 agonist in combination with the insulin therapy and also significantly less time spent in hypoglycemia when the GLP-1 agonist Liraglutide therapy was applied with insulin in T1DM patients with residual *β*-cell function [147, 148]. Therefore, lowering the time spent with the glucose levels below 3.9 mmol/L might be an advantage in the everyday clinical praxis because hypoglycaemia is still an existing problem.

Either the early immunization against the potential enhancers in a susceptible population that might be a realistic approach in prevention programs or the immune tolerance induction in combination with parallel therapies targeting *β*-cell recovery that might provide future alternatives for the therapy of the already developed disease will enter a highly competitive field where the already existing standard care provides T1DM patients with good quality of life and acceptable life expectancy [149].

## Figures and Tables

**Table 1:** T1DM-associated non-MHC risk polymorphisms (SNPs) with an odds ratio above 1.4 and the reported/mapped candidate genes in GWAStudies ranked by the highest corresponding odds ratio (OR) values in addition to the candidate genes (CTLA4, IFIH1, IL2, and PTPN2) that due to their immunological functions have been discussed in detail in the text.

Region	SNPs	Reported gene(s)	Mapped gene	Odds ratio	Risk allele frequency	*P* value	Gene product function [42]	Context	Initial sample size	Replication sample size	Platform (SNPs passing QC)	References
1p13.2	rs6679677	PHTF1, PTPN22	PHTF1-RSBN1	1,89	0,1	1*E* − 40	PHTF1: transcription factor; RSBN1: not fully characterized	Intergenic	1,963 cases, 2,938 cntrl	2997 trios, 4,000 cases, 5,000 cntrl	See [15]	[10]
	rs2476601	PTPN22	PTPN22	NR	NR	9*E* − 85	Lymphoid-specific intracellular phosphatase, a negative regulator of many signal transduction pathways	Missense	7,514 cases, 9,045 cntrl	4,267 cases, 4,670 cntrl, 4,342 trios	Affymetrix and illumina (841,622) (imputed)	[13]
				1,8	0,09	1*E* − 07		Missense	467 trios, 561 cases, 1,143 cntrl	2,350 individuals in 549 families; 390 trios	Illumina (543,071)	[16]
				1,98	0,09	2*E* − 80		Missense	1,963 cases, 2,938 cntrl	2997 trios, 4,000 cases, 5,000 cntrl	See [15]	[10]
	rs6679677		PHTF1-RSBN1	NR	NR	1*E* − 40		Intergenic	3,561 cases, 4,646 cntrl	6,225 cases, 6,946 cntrl, 3,064 trios	Affymetrix (up to 335,565)	[14]
				1,82	0,1	5*E* − 26		Intergenic	1,963 cases, 2,938 cntrl	See [10]	Affymetrix (469,557)	[15]

11p15.5	rs1004446	INS	IGF2; IGF2-AS; INS-IGF2	1,61	0,65	4*E* − 09	Insulin hormone, IGF-2 growth factor	Intron	467 trios, 561 cases, 1,143 cntrl	2,350 individuals in 549 families; 390 trios	Illumina (543,071)	[16]
	rs3741208		IGF2; IGF2-AS; INS-IGF2	1,25	0,38	2*E* − 07		Intron; ncRNA	1,963 cases, 2,938 cntrl	2997 trios, 4,000 cases, 5,000 cntrl	See [15]	[10]
	rs7111341		MIR4686-ASCL2	NR	NR	4*E* − 48	MIR4686: micro-RNA; ASCL2: a helix-loop-helix transcription factor involved in the determination of the neuronal precursors in the central and peripheral nervous system	Intergenic	7,514 cases, 9,045 cntrl	4,267 cases, 4,670 cntrl, 4,342 trios	Affymetrix and illumina (841,622) (imputed)	[13]

10p15.1	rs61839660	IL2RA	IL2RA	1,6	NR	5*E* − 09	IL2RA: as homodimer, a low-affinity receptor of IL-2; RPL32P23: pseudogene; RBM17: an RNA-binding protein, part of the spliceosome complex	Intron	16,179 Eu individuals	NR	NR (6,233,112) (imputed)	[12]
	rs12251307		RPL32P23-RBM17	NR	NR	1*E* − 13		Intergenic	7,514 cases, 9,045 cntrl	4,267 cases, 4,670 cntrl, 4,342 trios	Affymetrix and illumina (841,622) (imputed)	[13]
				NR	NR	2*E* − 06		Intergenic	3,561 cases, 4,646 cntrl	6,225 cases,6,946 cntrl, 3,064 trios	Affymetrix (up to 335,565)	[14]

12p13.31	rs3764021	NR	CLEC2D	1,57	0,47	5*E* − 08	C-type lectin, a member of the natural killer cell receptor family, inhibits osteoclast formation	Cds-synon	1,963 cases, 2,938 cntrl	See [10]	Affymetrix (469,557)	[15]

16p13.13	rs2903692	KIAA0350	CLEC16A	1,54	0,62	7*E* − 11	Member of the C-type lectin domain containing family	Intron	467 trios, 561 cases, 1,143 cntrl	2,350 individuals in 549 families; 390 trios	Illumina (543,071)	[16]
	rs12708716			1,19	0,65	5*E* − 07		Intron	1,963 cases, 2,938 cntrl	See [10]	Affymetrix (469,557)	[15]
				1,23	0,68	3*E* − 18		Intron	1,963 cases, 2,938 cntrl	2997 trios, 4,000 cases, 5,000 cntrl	See [15]	[10]

12p13.31	rs11052552	NR	NPM1P7-CLECL1	1,49	0,49	7*E* − 07	NPM1P7: pseudogene CLECL1: transmembrane, C-type lectin-like protein highly expressed on dendritic and B cells, may act as a T-cell costimulatory molecule	Intergenic	1,963 cases, 2,938 cntrl	See [10]	Affymetrix (469,557)	[15]

12q24.12	rs1265564	CUX2	CUX2	1,45	NR	1*E* − 16	A protein that contains three CUT domains and a homeodomain; both domains are DNA-binding motifs	Intron	16,179 eu individuals	NR	NR (6,233,112) (imputed)	[12]

13q22.2	rs539514	LMO7	LMO7	1,43	0,5	6*E* − 11	May be involved in protein-protein interactions	Intron	9,934 eu cases, 16,956 eu cntrl	1,120 eu affected trios	Affymetrix and illumina (~2.54 million) (imputed)	[17]

2q33.2	rs3087243	CTLA4	CTLA4	NR	NR	1*E* − 15	Transmits an inhibitory signal to T-cells	NearGene-3	7,514 cases, 9,045 cntrl	4,267 cases, 4,670 cntrl, 4,342 trios	Affymetrix and illumina (841,622) (imputed)	[13]
				NR	NR	8*E* − 11		NearGene-3	3,561 cases, 4,646 cntrl	6,225 cases, 6,946 cntrl, 3,064 trios	Affymetrix (up to 335,565)	[14]

2q24.2	rs1990760	IFIH1	IFIH1	NR	NR	7*E* − 11	Acts as a cytoplasmic sensor of viral nucleic acids	Missense	7,514 cases, 9,045 controls	4,267 cases, 4,670 controls, 4,342 trios	Affymetrix and illumina (841,622) (imputed)	[13]
				1,18	0,6	2*E* − 11		Missense	1,963 cases, 2,938 controls	2997 trios, 4,000 cases, 5,000 controls	See [15]	[10]

4q27	rs4505848	IL2	KIAA1109	NR	NR	5*E* − 13	KIAA1109: thought to function in the regulation of epithelial growth and differentiation and in tumor development	Intron	7,514 cases, 9,045 cntrl	4,267 cases, 4,670 cntrl, 4,342 trios	Affymetrix and illumina (841,622) (imputed)	[13]
	rs2069762	IL2	IL2	0,889	NR	NR	Cytokine, key activator of T-cells	Intron	8506 T1DM samples		Affymetrix and illumina (841,622) (imputed)	[29]

18p11.21	rs1893217	PTPN2	PTPN2	NR	NR	4*E* − 15	Member of the protein tyrosine phosphatase (PTP) family. Reported to have inhibitory role in beta-cell apoptosis	Intron	7,514 cases, 9,045 cntrl	4,267 cases, 4,670 cntrl, 4,342 trios	Affymetrix and illumina (841,622) (imputed)	[13]
	rs2542151		PSMG2-PTPN2	NR	NR	9*E* − 08	PSMG2: proteasome assembly chaperone	Intergenic	3,561 cases, 4,646 cntrl	6,225 cases, 6,946 cntrl, 3,064 trios	Affymetrix (up to 335,565)	[14]
				1,3	0,16	1*E* − 14		Intergenic	1,963 cases, 2,938 cntrl	2997 trios, 4,000 cases, 5,000 cntrl	See [15]	[10]

## Abbreviations

AIP: Autoimmune pancreatitis
APC: Antigen-presenting cell
BAFF: B-lymphocyte activating factor
BAK: BCL2-antagonist/killer
BAX: BCL2-associated X protein
BCL2: B-cell CLL/lymphoma 2
BCL2L11: BCL2-like 11
BCL-XL: BCL2-like protein 1
BH3: BCL2 homologous 3
CBV: Coxsackie-B virus
CD: Cluster of differentiation
CCL: Chemokine (C-C motif) ligand
CTLA-4: Cytotoxic T-lymphocyte antigen 4
CXCL: Chemokine (C-X-C motif) ligand
CXCR: C-X-C chemokine receptor
CMV: Cytomegalovirus
DC: Dendritic cell
DIPP: Diabetes prediction and prevention
DP5: Death protein 5
DPP-4: Dipeptidyl-peptidase-4
ERK: Extracellular signal-regulated kinase
Foxp3: Forkhead box P3
FT1DM: Fulminant type 1 diabetes
GAD: Glutamic acid decarboxylase
GKO: Gene knockout
gp: Glycoprotein
GWAStudies: Genomewide association Studies
HLA: Human leukocyte antigen
IDDM2: Insulin-dependent diabetes mellitus 2 locus
IFIH1: Interferon induced with helicase C domain 1
IFN: Interferon
IL: Interleukin
Ins: Insulin gene
iT_reg_: Induced regulatory T-cell
JNK1: c-Jun N-terminal protein kinase 1
LCMV: Lymphocytic choriomeningitis virus
lncRNA: Long noncoding RNA
LPS: Lipopolysaccharide
miRNA: Micro-RNA
MHC: Major histocompatibility complex
NF-*κ*B: Nuclear factor *κ*B
NOD: Nonobese diabetic
nT_reg_: Natural regulatory T-cell
PTPN: Protein tyrosine phosphatase, nonreceptor type
PUMA: p53 upregulated modulator of apoptosis
RAG: Recombination-activating gene
RIP-gp: Rat insulin promoter glycoprotein
SNP: Single-nucleotide polymorphism
STAT: Signal transducer and activator of transcription
T1DM: Type 1 diabetes mellitus
TCR: T-cell receptors
TGF: Transforming growth factor
Th: Helper T-cell
TLR: Toll-like receptor
TUNEL: Terminal deoxynucleotidyl transferase dUTP nick end labeling
Eu: Indicates participating individuals with European ancestry in the GWAStudies.
